# Prevalence of complications after surgery in treatment for cervical compressive myelopathy: A meta-analysis for last decade

**DOI:** 10.1097/MD.0000000000006421

**Published:** 2017-03-24

**Authors:** Tao Wang, Xiao-Ming Tian, Si-Kai Liu, Hui Wang, Ying-Ze Zhang, Wen-Yuan Ding

**Affiliations:** a Department of Spinal Surgery, The Third Hospital of Hebei Medical University; b Hebei Provincial Key Laboratory of Orthopedic Biomechanics, Shijiazhuang, China.

**Keywords:** cervical, complications, incidence, meta-analysis

## Abstract

**Purpose:**

We aim to perform a meta-analysis on prevalence of all kinds of operation-related complications following surgery treating cervical compressive myelopathy (CCM) and to provide reference for surgeons making surgical plan.

**Methods:**

An extensive search of literature was performed in PubMed/MEDLINE, Embase, the Cochrane library, CNKI, and WANFANG databases on incidence of operation-related complications from January 2007 to November 2016. Data was calculated and data analysis was conducted with STATA 12.0 and Revman 5.3.

**Results:**

A total of 107 studies included 1705 of 8612 patients (20.1%, 95% CI 17.3%–22.8%) on overall complications. The incidence of C5 plasy, cerebrospinal fluid (CSF), infection, axial pain, dysphagia, hoarseness, fusion failure, graft subsidence, graft dislodgment, and epidural hematoma is 5.3% (95% CI 4.3%–6.2%), 1.9% (95% CI 1.3%–2.4%), 2.8% (95% CI 1.7%–4.0%), 15.6% (95% CI 11.7%–19.5%), 16.8% (95% CI 13.6%–19.9%), 4.0% (95% CI 2.3%–5.7%), 2.6% (95% CI 0.2%–4.9%), 3.7% (95% CI 2.0%–5.5%), 3.4% (95% CI 2.0%–4.8%), 1.1% (95% CI 0.7%–1.5%), respectively. Patients with ossification of posterior longitudinal ligament (OPLL) (6.3%) had a higher prevalence of C5 plasy than those with cervical spondylotic myelopathy (CSM) (4.1%), and a similar trend in CSF (12.2% vs 0.9%). Individuals after laminectomy and fusion (LF) had highest rate of C5 plasy (15.2%), while those who underwent anterior cervical discectomy and fusion (ACDF) had the lowest prevalence (2.0%). Compared with patients after other surgical options, individuals after anterior cervical corpectomy and fusion (ACCF) have the highest rate of CSF (4.2%), infection (14.2%), and epidural hematoma (3.1%). Patients after ACDF (4.8%) had a higher prevalence of hoarseness than those with ACCF (3.0%), and a similar trend for dysphagia between anterior corpectomy combined with discectomy (ACCDF) and ACCF (16.8% vs 9.9%).

**Conclusions:**

Based on our meta-analysis, patients with OPLL have a higher incidence of C5 palsy and CSF. Patients after LF have a higher incidence of C5 palsy, ACCDF have a higher incidence of dysphagia, ACCF have a higher incidence of CSF and infection and ACDF have a higher incidence of hoarseness. These figures may be useful in the estimation of the probability of complications following cervical surgery.

## 1 Introduction

Cervical compressive myelopathy (CCM), caused by cervical spondylotic myelopathy (CSM) or ossification of posterior longitudinal ligament (OPLL), is a common cervical degenerative disease with increasing elder population, seriously impacting quality of life and even leading to disability.^[1–3]^ The aim of surgery is to decompress spinal cord and preserve the stability of the spinal column.^[3–7]^ However, the selection of optimal surgical treatment for CCM remains controversial.^[4,5,8–11]^ Surgeries, widely used in clinic mainly involved anterior and posterior approaches, including anterior cervical discectomy and fusion (ACDF), anterior cervical corpectomy and fusion (ACCF), anterior corpectomy combined with discectomy (ACCDF), laminoplasty (LP), and laminectomy with fusion (LF).^[10–16]^ Each approach has its own advantages and disadvantages. Anterior approaches are propitious to solve pathogenic structures from anterior, but it has a high risk of complications, like dysphagia, hoarseness, or artery injury, as reported by previous studies.^[3–5,7,9]^ Posterior approaches could adequately decompress spinal cord, but it was reported that posterior approaches were more likely to cause C5 plasy and cervical kyphosis.

Even though, many studies reported on surgical selection for CCM. But there is no meta-analysis on prevalence of complications following cervical surgery treating for CCM. The purpose of our study is to explore incidence of operation-related complications after cervical surgery and we hope that it is helpful in the estimation of the probability of complications following cervical surgery.

## 2 Materials and methods

### 2.1 Ethics statement

There is no need to seek informed consent from patients, since this is a meta-analysis based on the published data, without any potential harm to the patients; this is approved by Ethics Committee of The Third Hospital of HeBei Medical University.

### 2.2 Search strategy

An extensive search of literature was performed in PubMed/MEDLINE, Embase, the Cochrane library, CNKI, and WANFANG databases. The following key words were used for search: “complications,” “cervical,” “C5 plasy,” “CSF,” “infection,” “axial pain,” “dysphagia,” “hoarseness,” “fusion failure,” “graft subsidence,” “graft dislodgment,” “epidural hematoma,” “anterior cervical discectomy and fusion,” “anterior cervical corpectomy and fusion,” “corpectomy combined with discectomy,” “laminoplasty,” “laminectomy and fusion,” “cervical spondylotic myelopathy,” and “ossification of posterior longitudinal ligament” from January 2007 to November 2016, with various combinations of the operators “AND” and “OR.” Language was restricted to Chinese and English.

### 2.3 Inclusion criteria

Studies were included if they met the following criteria: randomized or nonrandomized controlled study; age greater than or equal to 18 years old; studies on complications after cervical surgery.

### 2.4 Exclusion criteria

Studies were excluded if they met the following criteria: had repeated data; did not report outcomes of interest; in vitro human cadaveric biomechanical studies; earlier trial, reviews, and case-reports; sample size >1000 or <30; CCM caused by trauma or tumour; have a history of cervical surgery.

### 2.5 Selection of studies

Two reviewers independently reviewed all subjects, abstracts, and the full text of articles. Then the eligible trials were selected according to the inclusion criteria. When consensus could not be reached, a third reviewer was consulted to resolve the disagreement.

### 2.6 Data extraction and management

Two reviewers extracted data independently. The data extracted including the following categories: study ID, study design, study location, number of total patients and patients with complications, diagnose, complications category, incidence of complications after anterior or posterior approaches including ACDF, ACCF, ACCDF, LP, and LF.

### 2.7 Statistical analysis

Data analysis was performed with STATA 12.0 (Stata Corporation, College Station, TX). Both were reported with 95% confidence intervals (CI), and a *P* value of 0.05 was used as the level of statistical significance. Assessment for statistical heterogeneity was calculated using the *I*^2^ tests, which described the proportion of the total variation in meta-analysis assessments from 0% to 100%. The random effects model was used for the analysis when an obvious heterogeneity was observed among the included studies (*I*^2^ >50%). The fixed-effects model was used when there was no significant heterogeneity between the included studies (*I*^2^ ≤50%).^[17,18]^ Flow diagram was performed with Revman 5.3

### 2.8 Test for risk of publication bias

We performed a visual inspection of the funnel plot for publication bias. The funnel plot should be asymmetric when there is publication bias and symmetric in the case of no publication bias. We performed Egger and Begg tests to measure the funnel plot asymmetry by using a significance level of *P*<0.05. The trim and fill computation was used to estimate the effect of publication bias.

## 3 RESULTS

### 3.1 Search results

**Figure 1:**
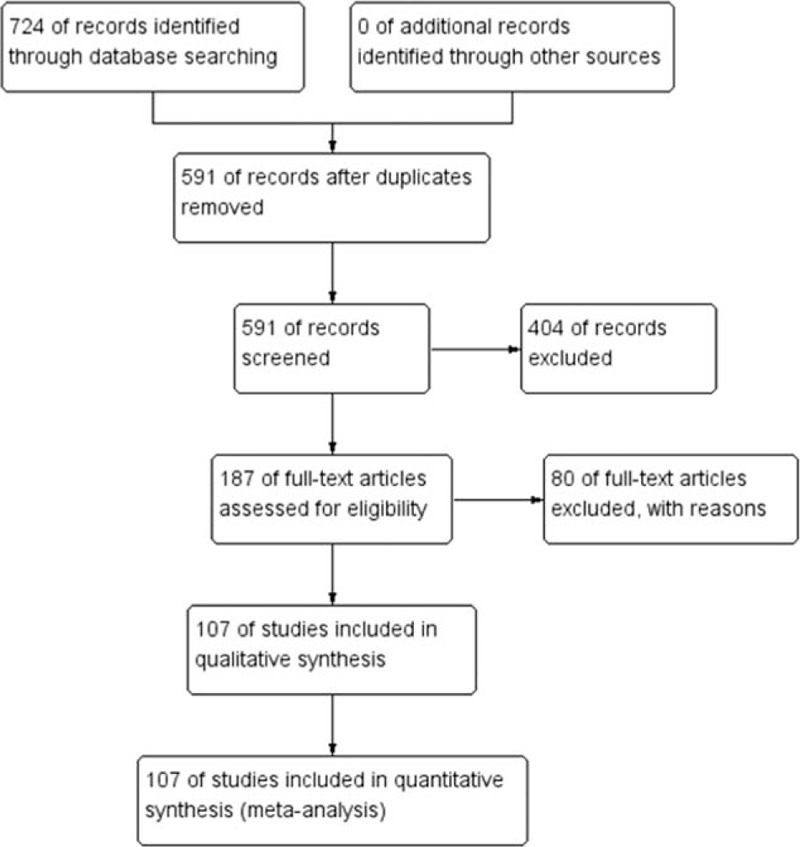
Flow diagram of study selection.

We had searched 631 English studies in MEDLINE, EMBASE, 93 Chinese studies in WANFANG and CNKI. Of these, 103 English articles and 30 Chinese articles after duplicates removed, 368 English articles and 36 Chinese articles were excluded due to unrelated studies. Seventy-three English articles and 7 Chinese articles were excluded due to eligibility criteria. As a result, a total of 107 studies were identified for this meta-analysis. The literature search procedure is shown in Fig. 1.

### 3.2 Baseline characteristics and quality assessment

A total of 8612 patients from 75 studies on total complications, 6349 patients from 57 studies on C5 plasy, 5007 patients from 36 studies on CSF, 591 patients from 6 studies on graft subsidence, 1102 patients from 10 studies on graft dislodgment, 2234 patients from 19 studies on hoarseness, 3489 patients from 25 studies on infection, 5841 patients from 38 studies on dysphagia, 689 patients from 5 studies on fusion failure, 2185 patients from 14 studies on epidural hematoma, 2650 patients from 23 studies on axial pain were included in our study. Table 1   shows the baseline characteristics of included articles.

**Table 1:**
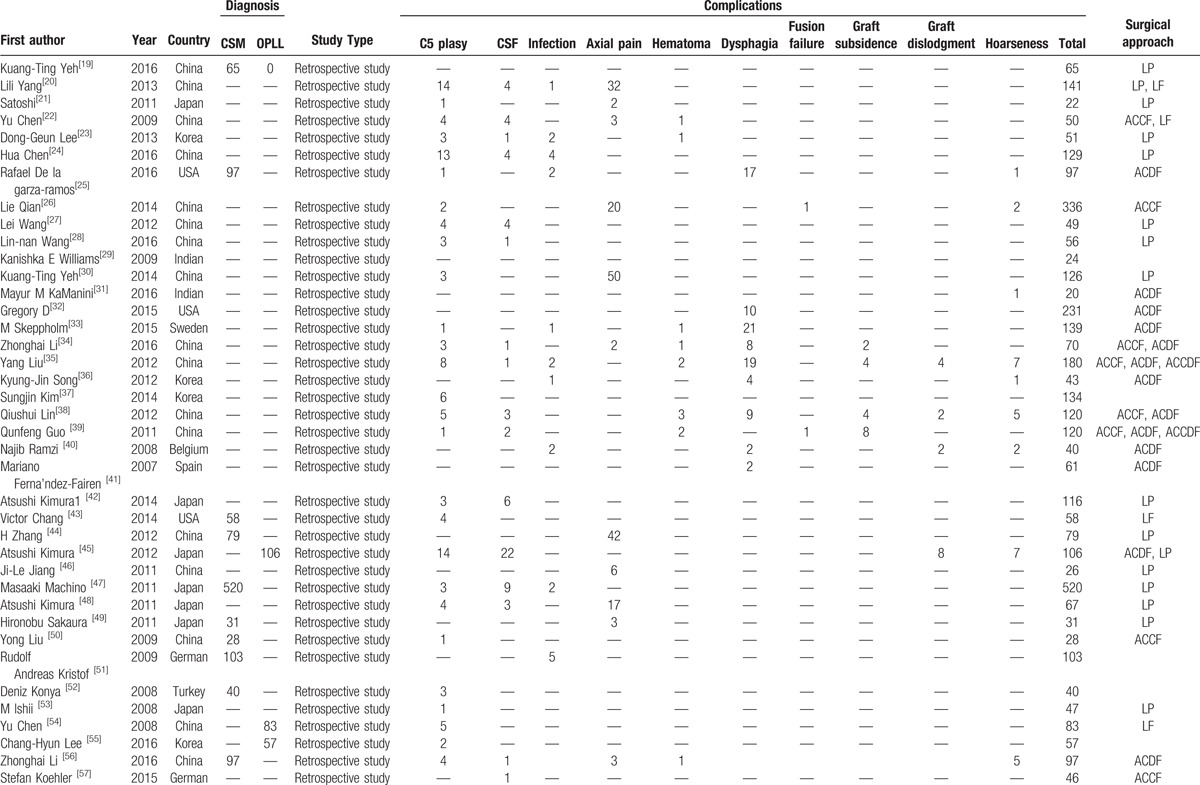
Characteristics of included studies.

**Table 1 (Continued):**
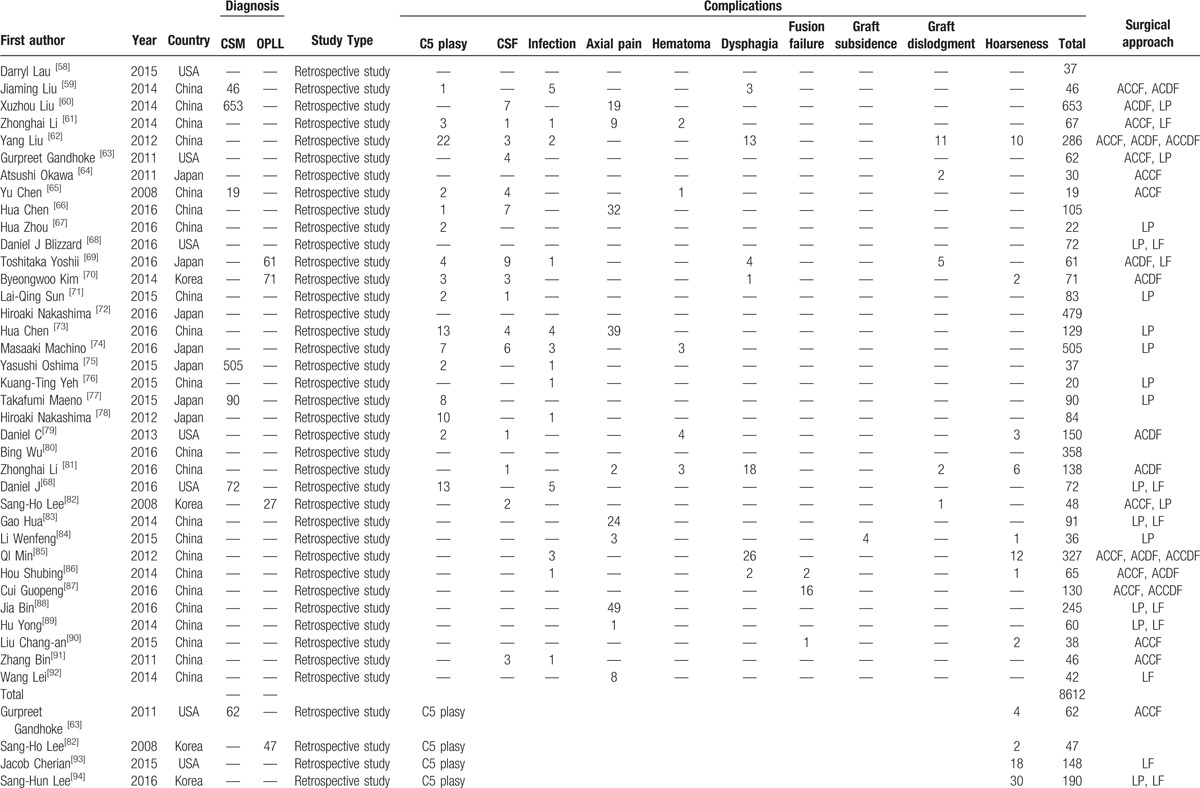
Characteristics of included studies.

**Table 1 (Continued):**
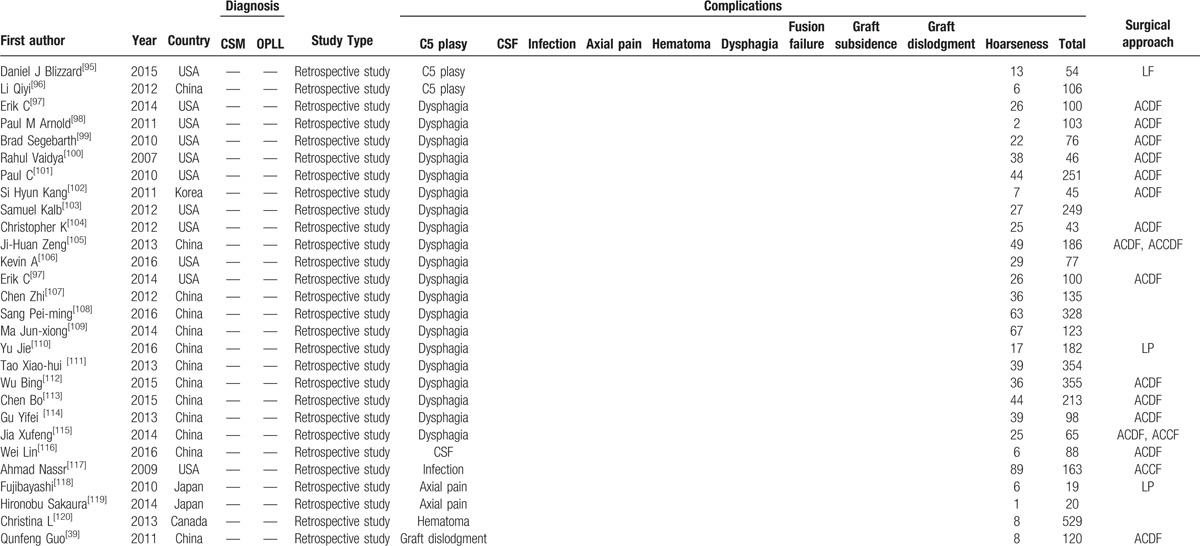
Characteristics of included studies.

All included studies were retrospective studies, Newcastle Ottawa Quality Assessment Scale (NOQAS) was applied to estimate the quality of each study. We used NOQAS, the maximum of 9 points, to assess quality of selection for nonrandomized case controlled studies and cohort studies in terms of comparability, exposure, and outcomes. Among these studies, 95 studies scored 8 points and 3 studies scored 12 points. Therefore, each study has relatively high quality (Table 2 ).

**Table 2:**
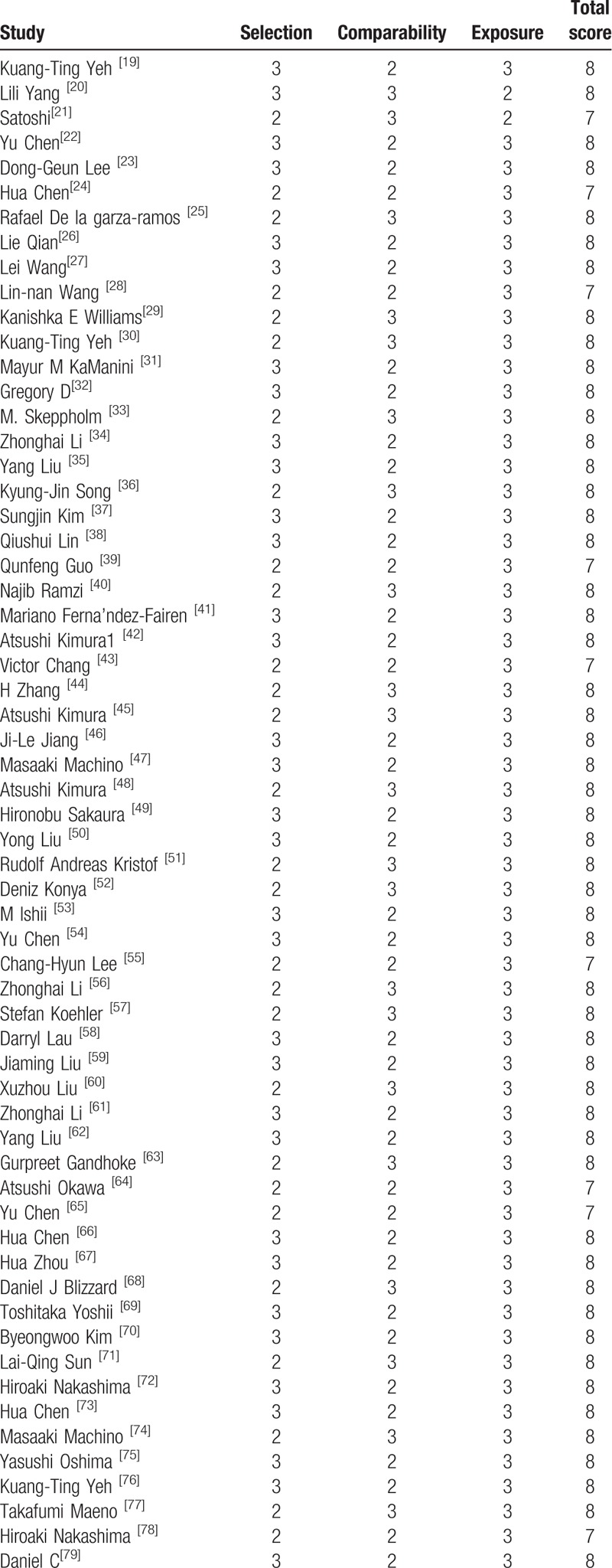
The quality assessment according to the Newcastle Ottawa Quality Assessment Scale (NOQAS) of each study.

**Table 2 (Continued):**
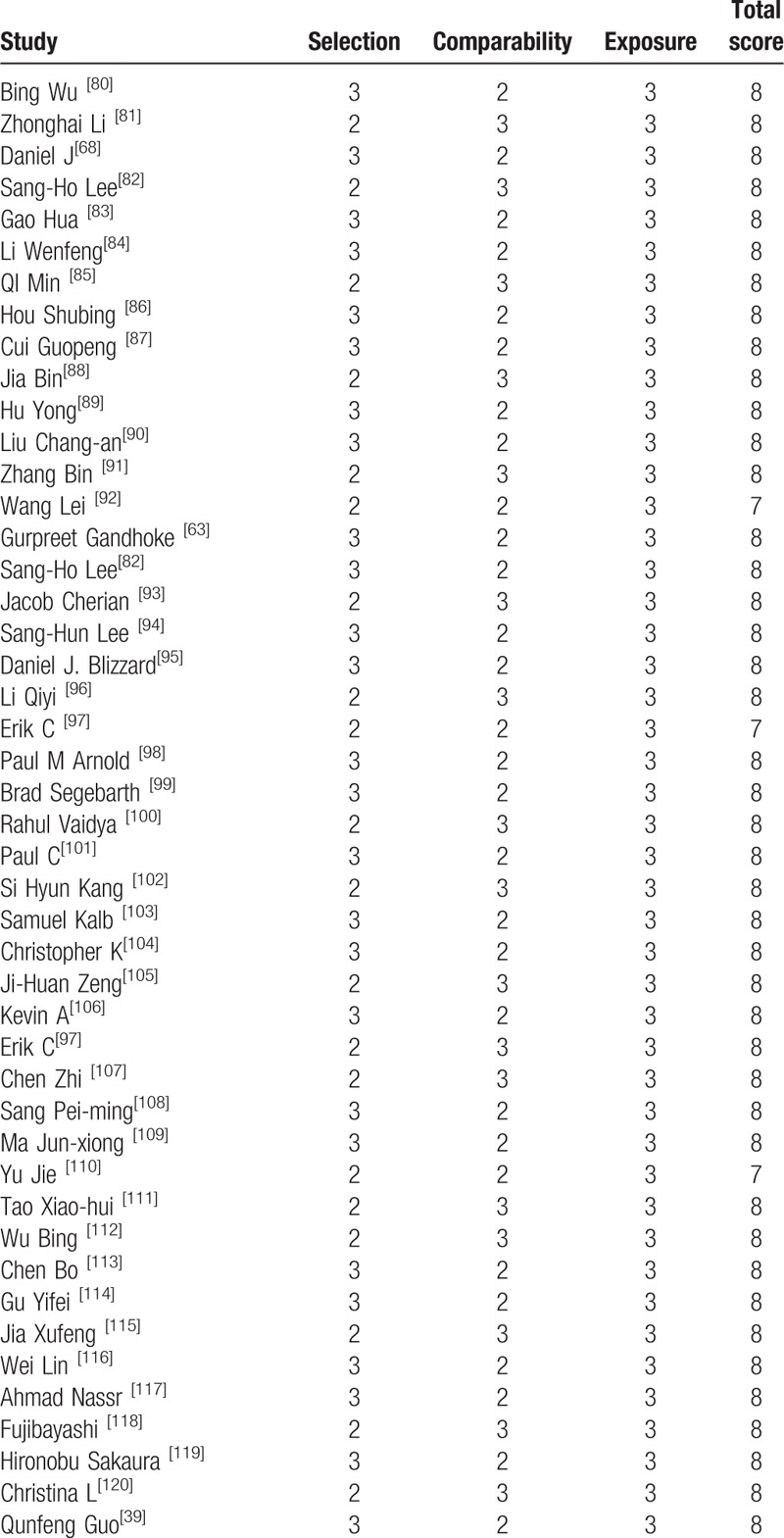
The quality assessment according to the Newcastle Ottawa Quality Assessment Scale (NOQAS) of each study.

### 3.3 Prevalence of overall complications

**Figure 2:**
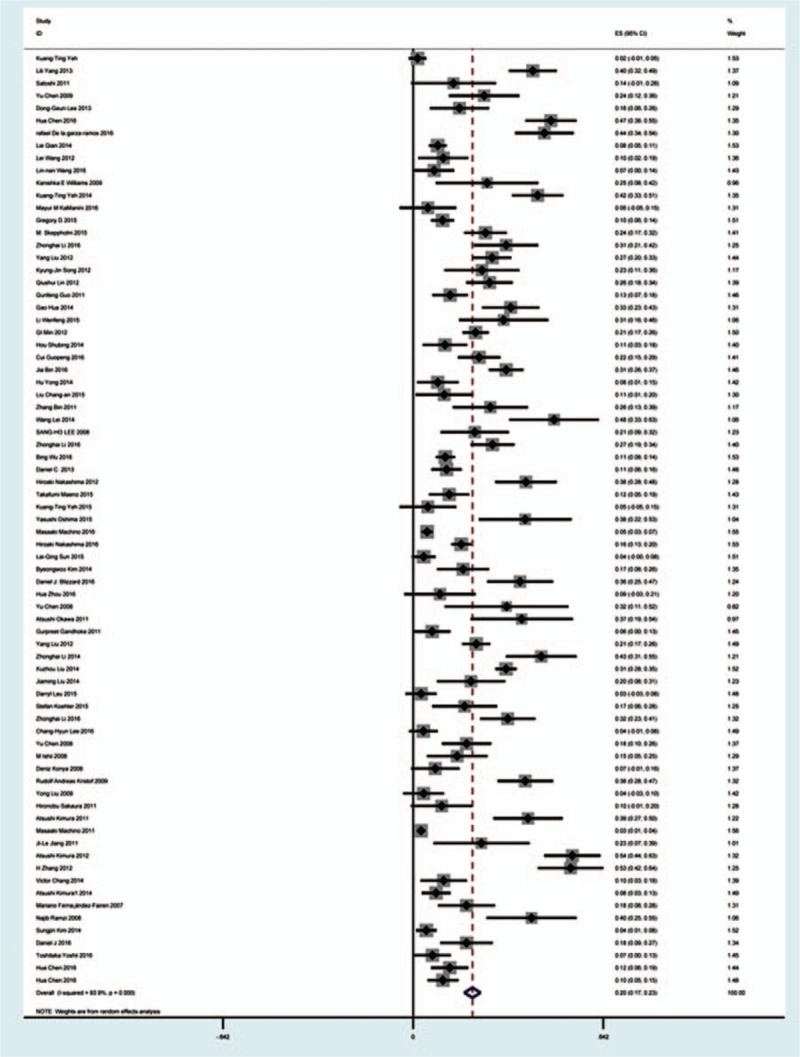
Forest plot showing incidence of overall complications after cervical surgery. CI = confidence interval, df = degrees of freedom, M–H = Mantel–Haenszel.

Seventy-five studies^[19–92]^ containing 1705 patients with overall complications of 8612 patients after cervical surgery were included. Figure 2 shows that the incidence was 20.1% (95% CI 17.3%–22.8%), with substantial heterogeneity of incidence observed. The incidence varied between 2.6% and 58.1%.

### 3.4 C5 plasy

**Figure 3:**
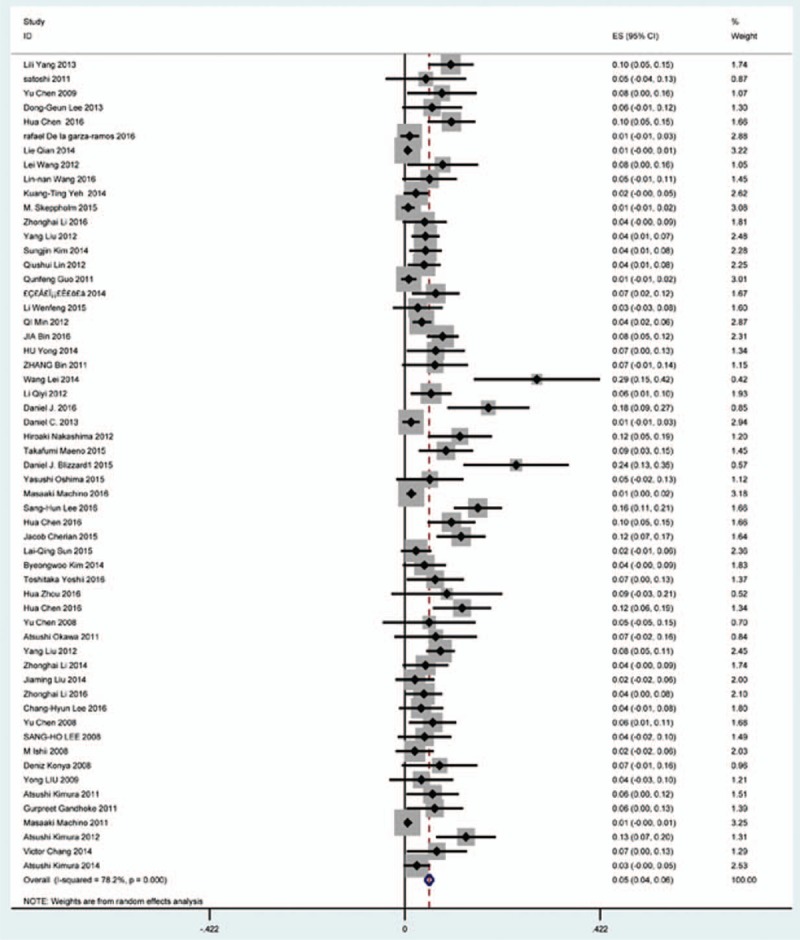
Forest plot showing incidence of C5. CI = confidence interval, df = degrees of freedom, M–H = Mantel–Haenszel.

**Figure 4:**
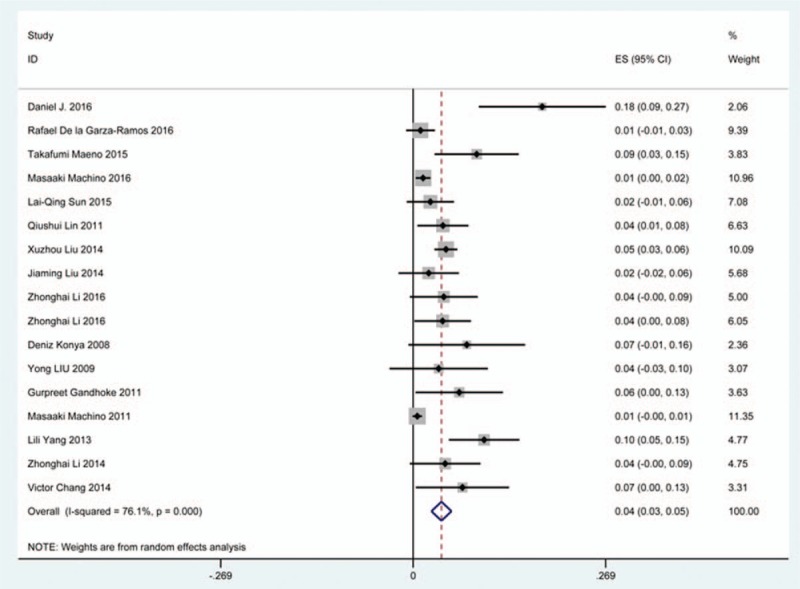
Forest plot showing incidence of C5 for patients with CSM. CI = confidence interval, CSM = cervical spondylotic myelopathy, df = degrees of freedom, M–H = Mantel–Haenszel.

**Figure 5:**
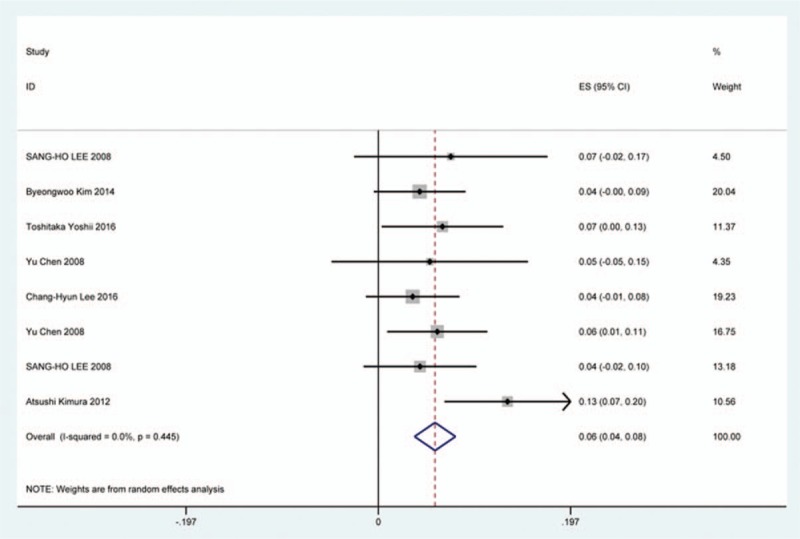
Forest plot showing incidence of C5 for patients with OPLL. CI = confidence interval, df = degrees of freedom, M–H = Mantel–Haenszel, OPLL = ossification of posterior longitudinal ligamen.

**Figure 6:**
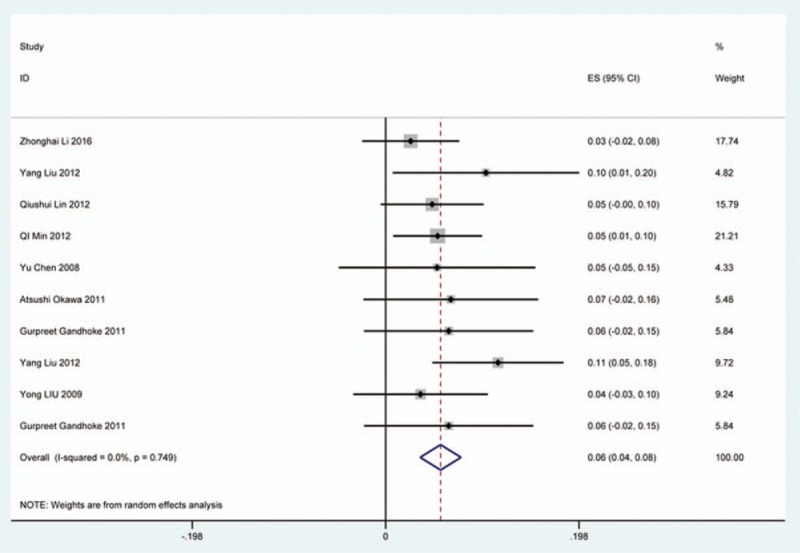
Forest plot showing incidence of C5 after ACCF. ACCF = anterior cervical corpectomy and fusion, CI = confidence interval, df = degrees of freedom, M–H = Mantel–Haenszel.

**Figure 7:**
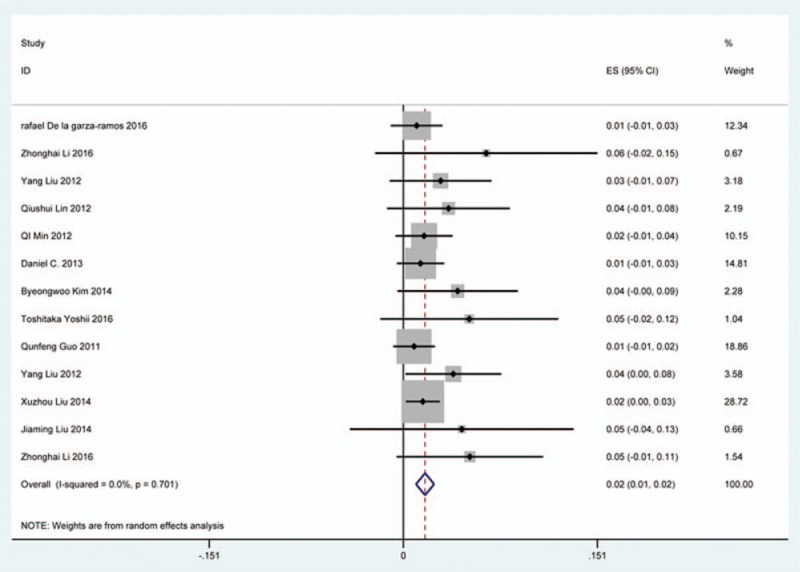
Forest plot showing incidence of C5 after ACDF. ACDF = anterior cervical discectomy and fusion, CI = confidence interval, df = degrees of freedom, M–H = Mantel–Haenszel.

**Figure 8:**
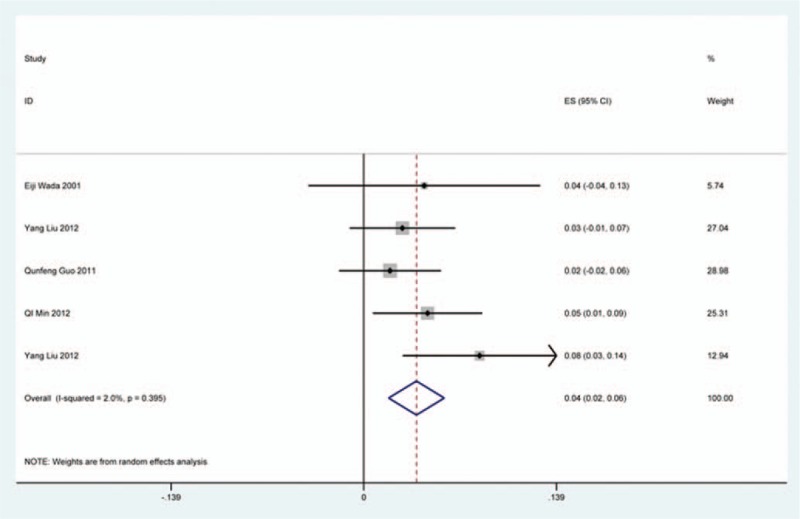
Forest plot showing incidence of C5 after ACCDF. ACCDF = anterior corpectomy combined with discectomy, CI = confidence interval, df = degrees of freedom, M–H = Mantel–Haenszel.

**Figure 9:**
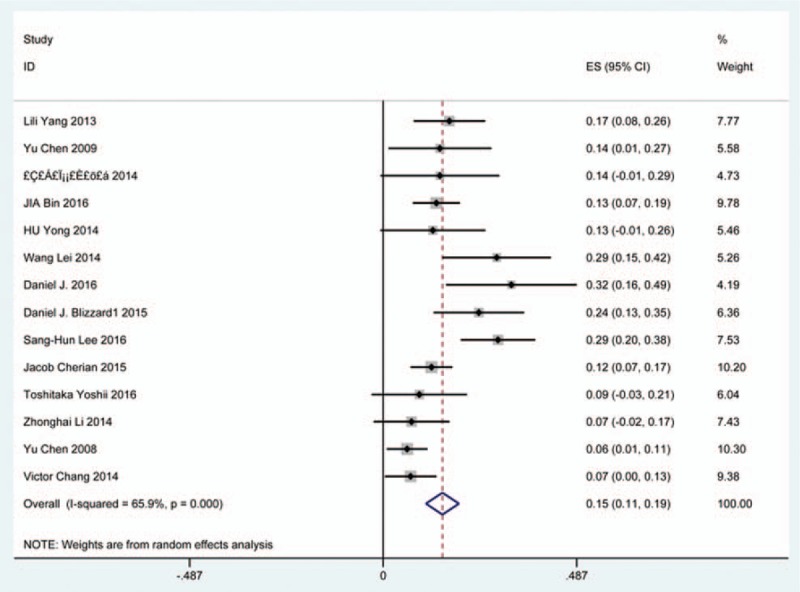
Forest plot showing incidence of C5 after LP. CI = confidence interval, df = degrees of freedom, LP = laminoplasty, M–H = Mantel–Haenszel.

**Figure 10:**
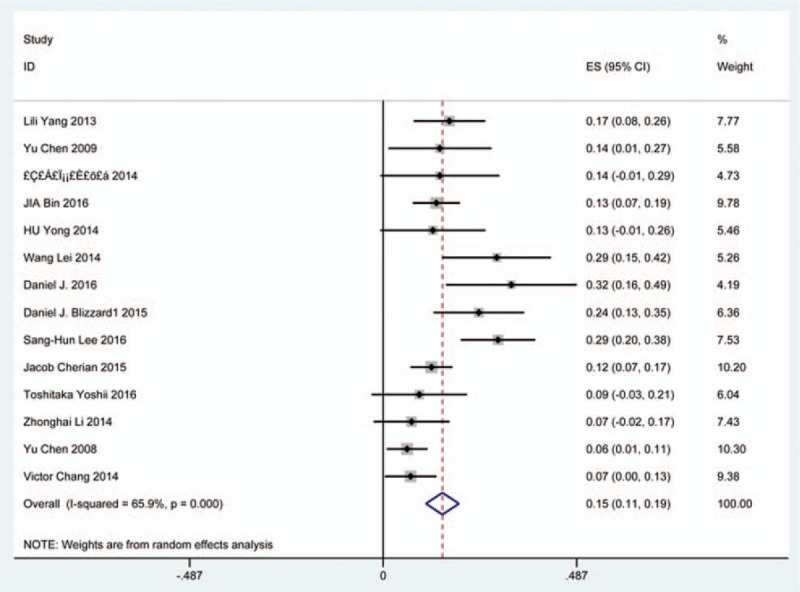
Forest plot showing incidence of C5 after LF. CI = confidence interval, df = degrees of freedom, LF = laminectomy and fusion, M–H = Mantel–Haenszel.

Fifty-seven studies^[20–28,30,33–35,37–39,42–43,45,47–48,50,52–56,59,61–71,73–75,77–79,82–85,88,89,91–96]^ containing 355 patients with C5 plasy of 6349 patients after cervical surgery were included. Figure 3 shows that the incidence was 5.3% (95% CI 4.3%–6.2%), with substantial heterogeneity of incidence observed. The incidence varied between 0.6% and 28.6%. Compared with patients with CSM (4.1%, 95% CI 2.9%–5.2%), patients with OPLL (6.3%, 95% CI 2.4%–5.2%) has a higher incidence (Figs. 4, 5). In terms of surgical methods, patients who underwent LP had the highest rate (15.2%, 95% CI 10.9%–19.1%), while those who received ACDF had the lowest rate (2.0%, 95% CI 0.8%–2.4%) (Fig 6, Fig 7, Fig 8, Fig 9, Fig 10).

### 3.5 Dysphagia

**Figure 11:**
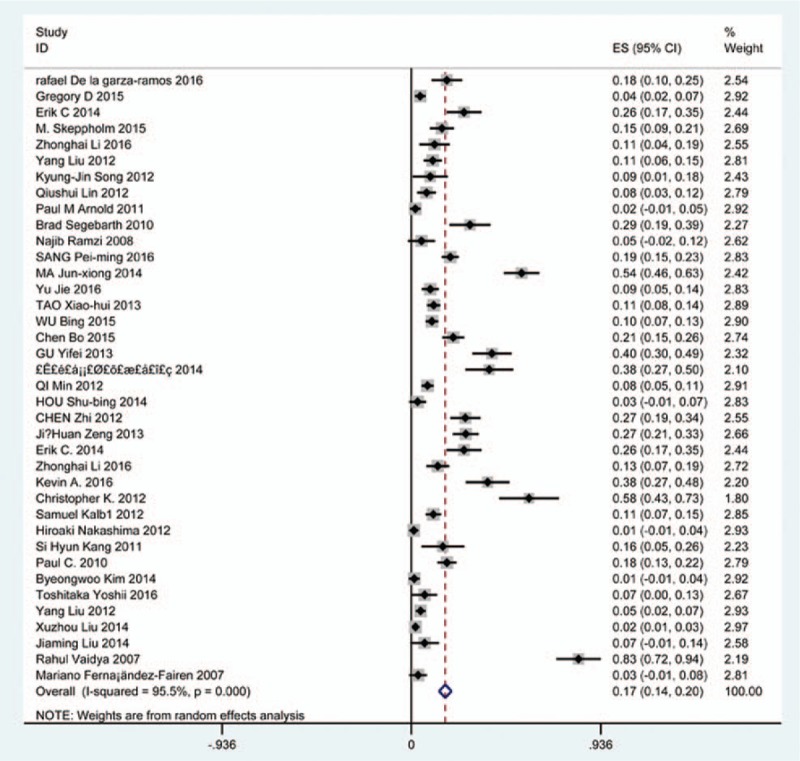
Forest plot showing incidence of dysphagia. CI = confidence interval, df = degrees of freedom, M–H = Mantel–Haenszel.

**Figure 12:**
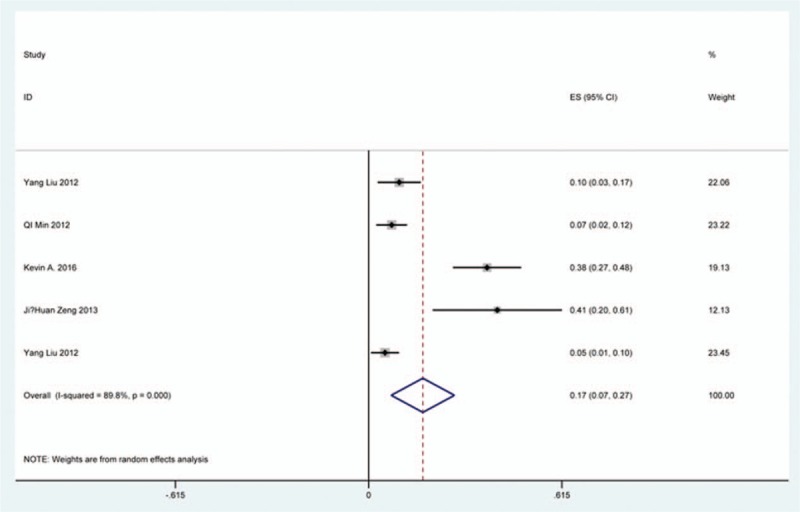
Forest plot showing incidence of dysphagia after ACCDF. ACCDF = anterior corpectomy combined with discectomy, CI = confidence interval, df = degrees of freedom, M–H = Mantel–Haenszel.

**Figure 13:**
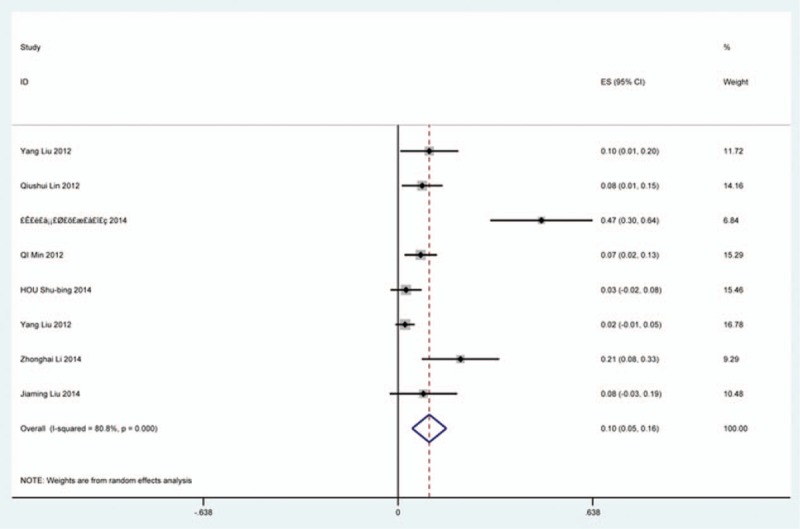
Forest plot showing incidence of dysphagia after ACCF. ACCF = anterior cervical corpectomy and fusion, CI = confidence interval, df = degrees of freedom, M–H = Mantel–Haenszel.

**Figure 14:**
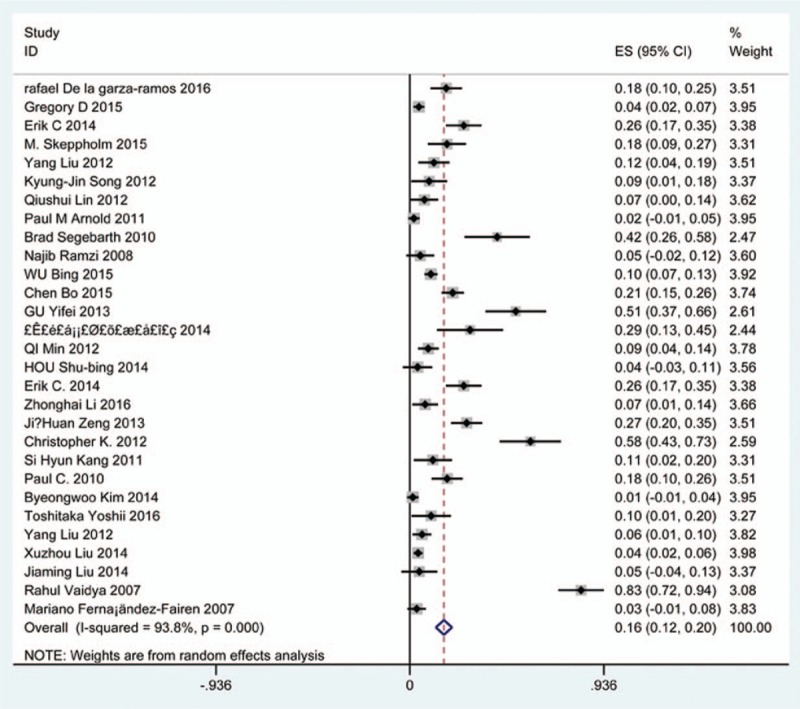
Forest plot showing incidence of dysphagia after ACDF. ACDF = anterior cervical discectomy and fusion, CI = confidence interval, df = degrees of freedom, M–H = Mantel–Haenszel.

Thirty-eight studies^[25,32–36,38,40–41,59–60,62,69–70,78,81,85,86,97–115]^ containing 835 patients with dysphagia of 5841 patients after cervical surgery were included. Figure 11 shows that the incidence was 16.8% (95% CI 13.6%–19.9%), with substantial heterogeneity of incidence observed. The incidence varied between 1.4% and 58.1%. Incidence for patients who underwent ACCDF and ACDF was 16.8% (95% CI 6.9%–27.2%) and 16.2% (95% CI 11.7%–19.8%), which is higher than those who received ACCF 9.9% (95% CI 4.8%–15.9%) (Fig 12, Fig 13, Fig 14).

### 3.6 Cerebrospinal fluid

**Figure 15:**
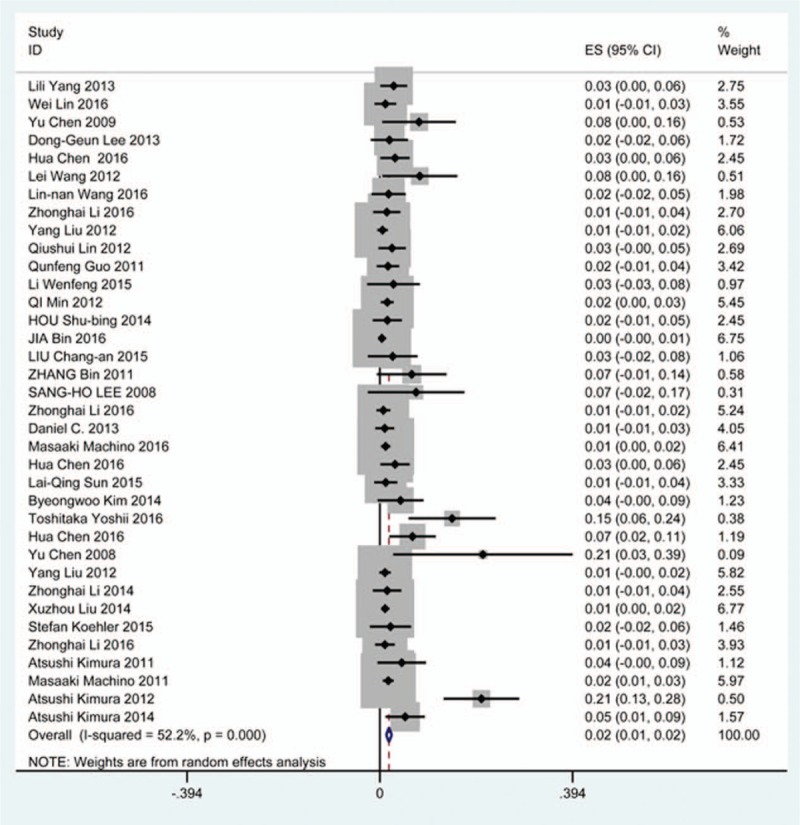
Forest plot showing incidence of CSF. CI = confidence interval, df = degrees of freedom, M–H = Mantel–Haenszel.

**Figure 16:**
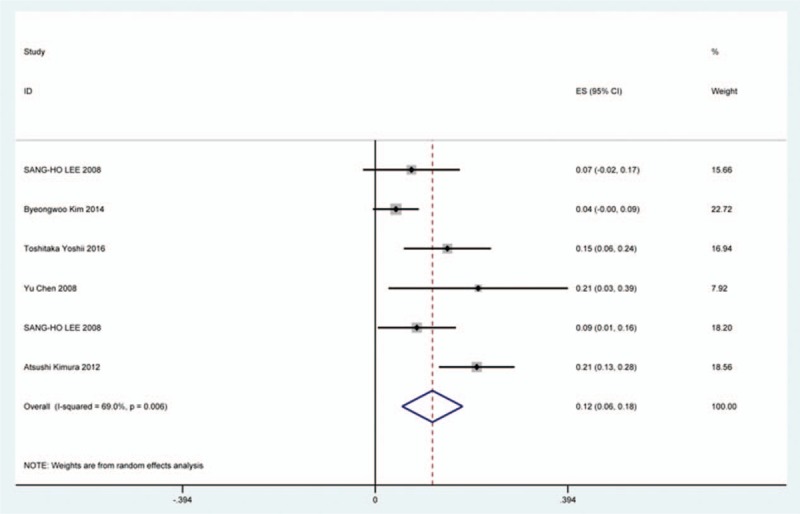
Forest plot showing incidence of CSF for patients with OPLL. CI = confidence interval, df = degrees of freedom, M–H = Mantel–Haenszel, OPLL = ossification of posterior longitudinal ligamen.

**Figure 17:**
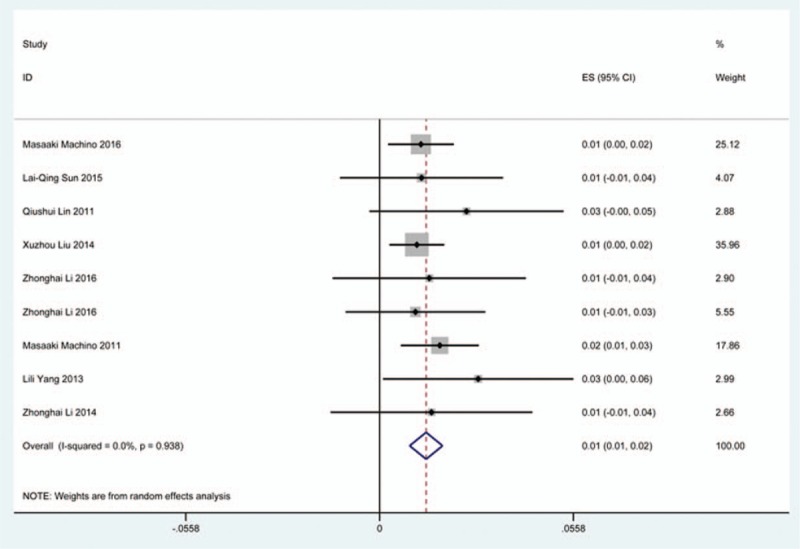
Forest plot showing incidence of CSF for patients with CSM. CI = confidence interval, CSM = cervical spondylotic myelopathy, df = degrees of freedom, M–H = Mantel–Haenszel.

**Figure 18:**
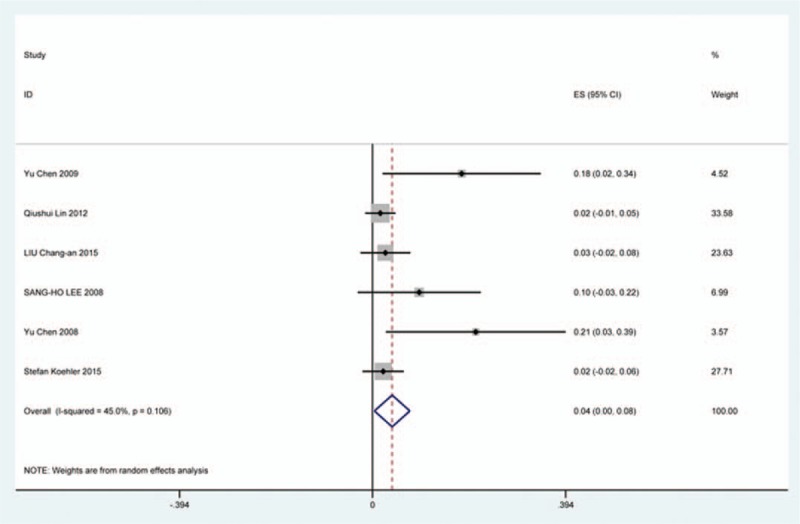
Forest plot showing incidence of CSF after ACCF. ACCF = anterior cervical corpectomy and fusion, CI = confidence interval, df = degrees of freedom, M–H = Mantel–Haenszel.

**Figure 19:**
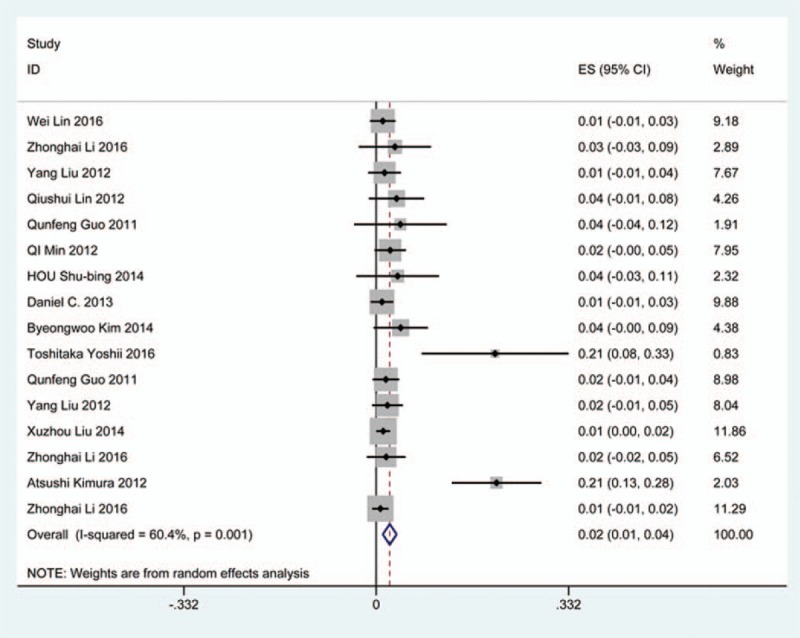
Forest plot showing incidence of CSF after ACDF. ACDF = anterior cervical discectomy and fusion, CI = confidence interval, df = degrees of freedom, M–H = Mantel–Haenszel.

**Figure 20:**
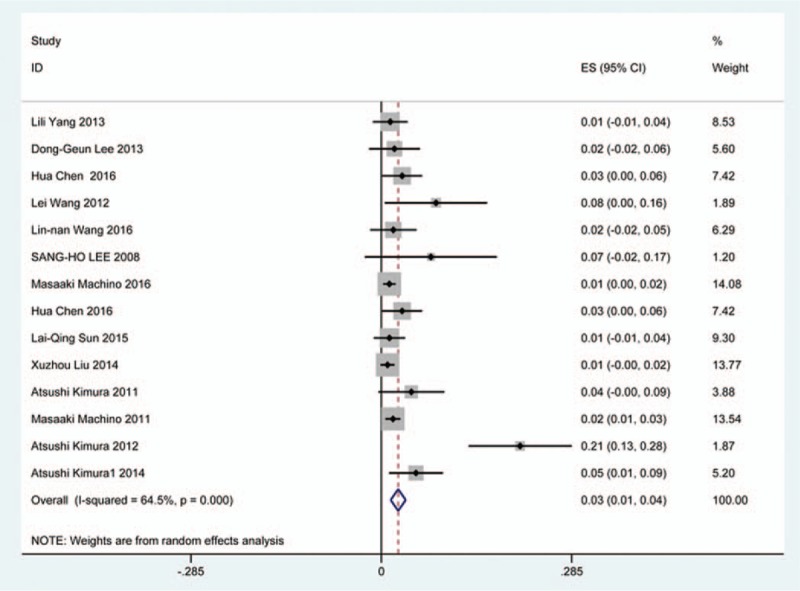
Forest plot showing incidence of CSF after LP. CI = confidence interval, df = degrees of freedom, LP = laminoplasty, M–H = Mantel–Haenszel.

Thirty-six studies^[20,22–24,27,28,34,35,38,39,42,45,47,48,56,57,60–62,65,66,69–71,73,74,79,81,82,84–86,88,90,91,116]^ containing 129 patients with CSF of 5007 patients after cervical surgery were included. Figure 15 shows that the incidence was 1.9% (95% CI 1.3%–2.4%), with substantial heterogeneity of incidence observed. The incidence varied between 0.4% and 21.1%. Compared with patients with CSM (0.9%, 95% CI 0.6%–1.7%), patients with OPLL (12.2%, 95% CI 6.2%–17.8%) have a higher incidence (Figs. 16, 17). As for surgical methods, patients who underwent ACCF had the highest rate (4.2%, 95% CI 0.3%–8.2%), while those who received ACDF had the lowest rate (1.9%, 95% CI 0.9%–4.0%) (Figs. 18–20 Fig 18, Fig 19, Fig 20).

### 3.7 Infection

**Figure 21:**
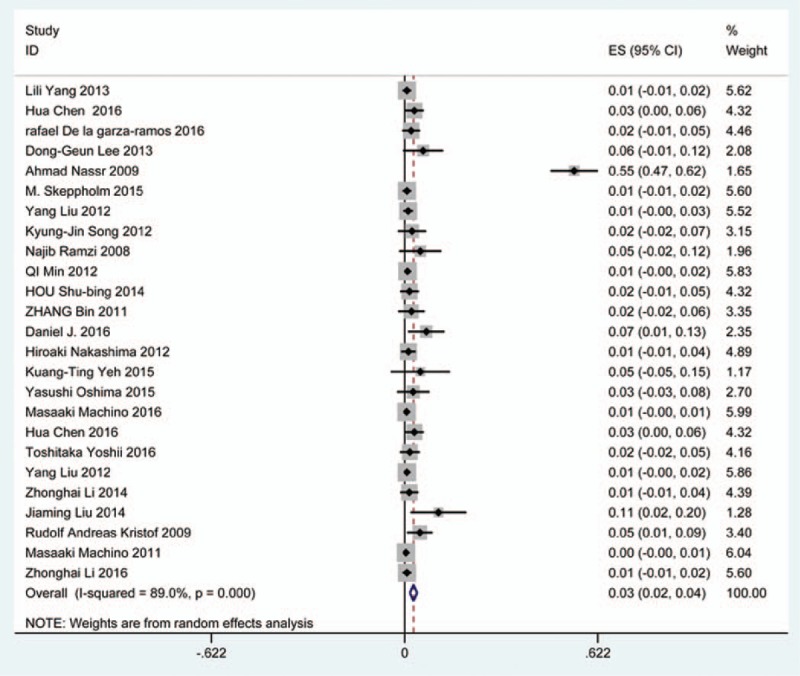
Forest plot showing incidence of infection. CI = confidence interval, df = degrees of freedom, M–H = Mantel–Haenszel.

**Figure 22:**
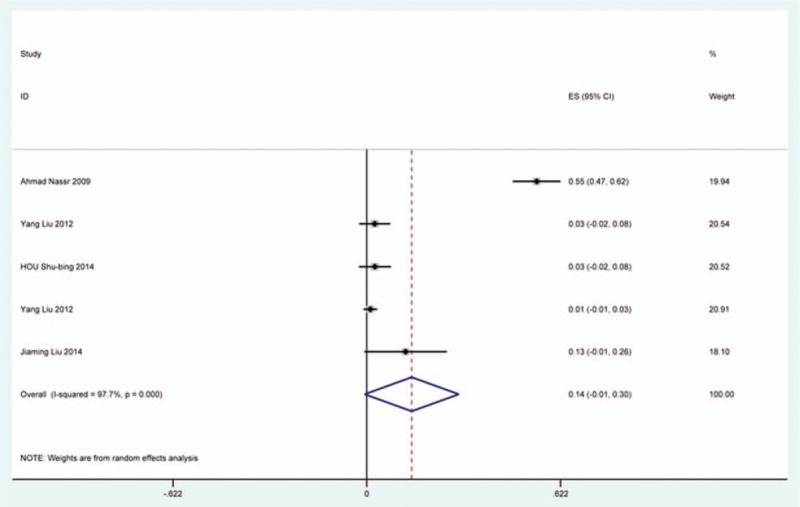
Funnel plot showing incidence of infection after ACCF. ACCF = anterior cervical corpectomy and fusion, CI = confidence interval, df = degrees of freedom, M–H = Mantel–Haenszel.

**Figure 23:**
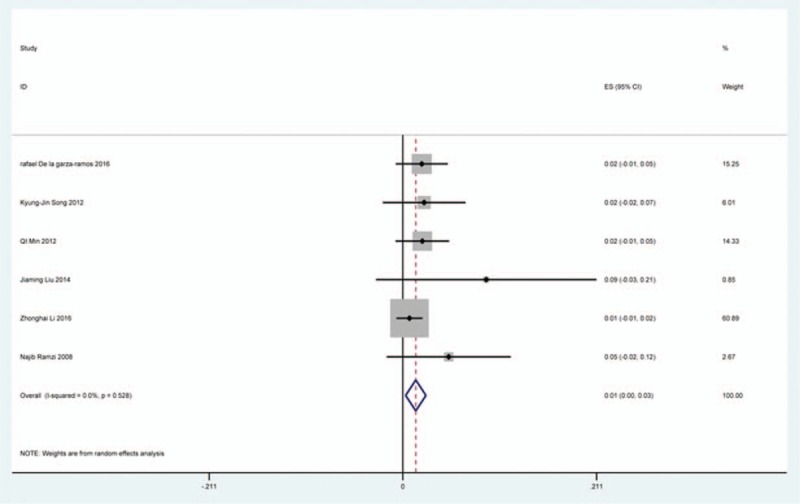
Forest plot showing incidence of Infection after ACDF. ACDF = anterior cervical discectomy and fusion, CI = confidence interval, df = degrees of freedom, M–H = Mantel–Haenszel.

**Figure 24:**
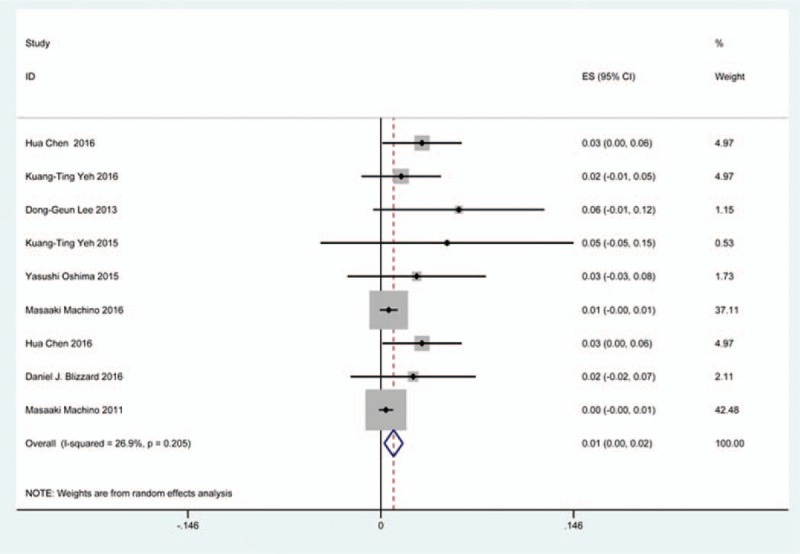
Forest plot showing incidence of infection after LP. CI = confidence interval, df = degrees of freedom, LP = laminoplasty, M–H = Mantel–Haenszel.

Twenty-five studies^[20,23–25,33,35,36,40,47,51,59,61,62,68,69,73–76,78,85,86,91,117,118]^ containing 142 patients with infection of 3489 patients after cervical surgery were included. Figure 21 shows that the incidence was 2.8% (95% CI 1.7%–4.0%), with substantial heterogeneity of incidence observed. The incidence varied between 0.4% and 54.6%. Incidence for the patients who underwent ACCF was 14.2% (95% CI −1.1%–30.3%), which higher than those who received ACDF (0.9%, 95% CI 0.2%–2.8%) and LP (2.1%, 95% CI 0.9%–3.2%) (Fig 22, Fig 23, Fig 24).

### 3.8 Axial pain

**Figure 25:**
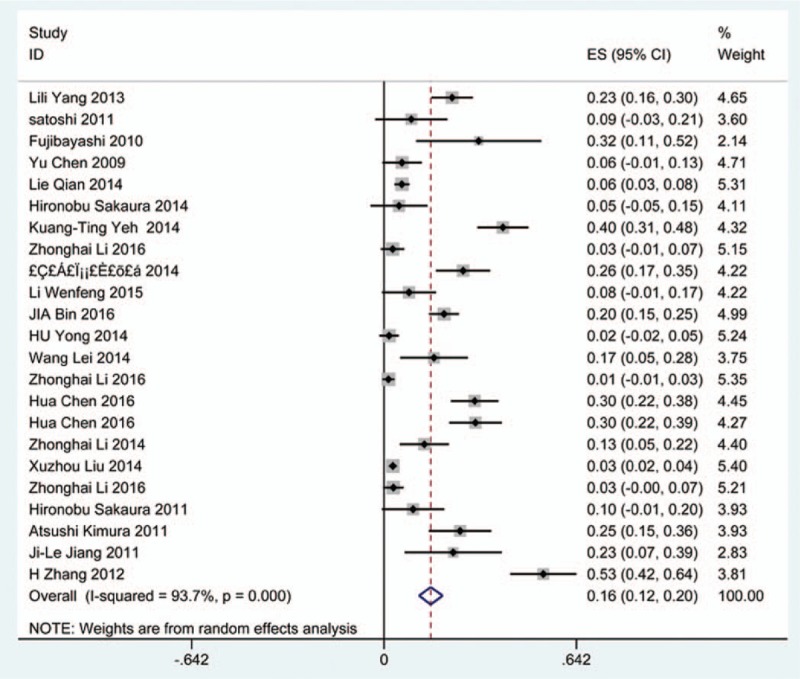
Forest plot showing incidence of axial pain. CI = confidence interval, df = degrees of freedom, M–H = Mantel–Haenszel.

**Figure 26:**
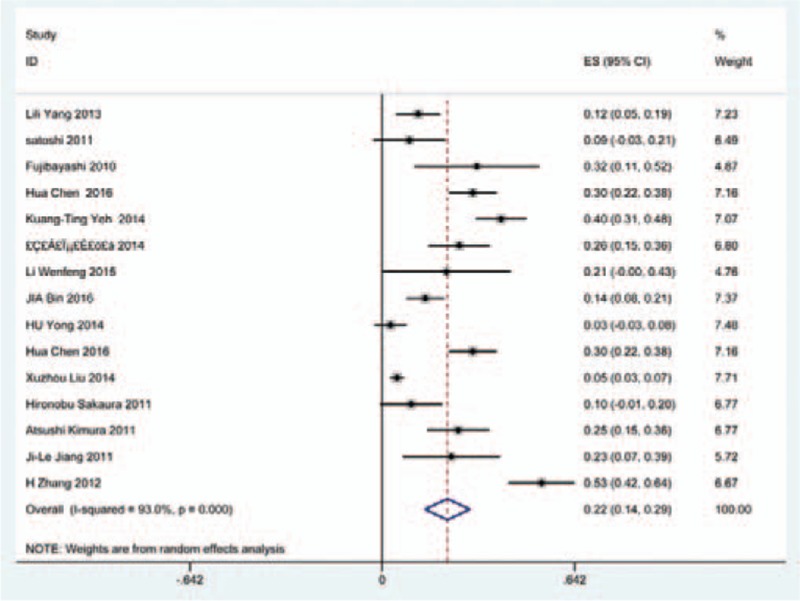
Forest plot showing incidence of axial pain after LP. CI = confidence interval, df = degrees of freedom, LP = laminoplasty, M–H = Mantel–Haenszel.

**Figure 27:**
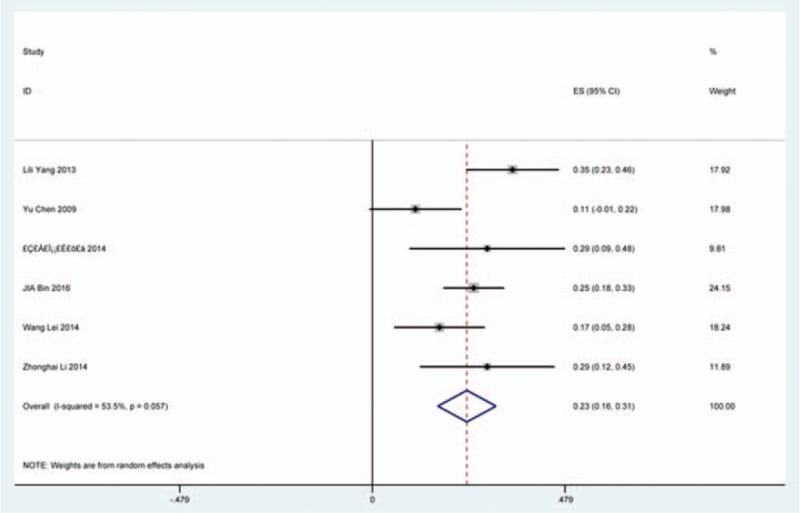
Forest plot showing incidence of axial pain after LF. df = degrees of freedom, I = confidence interval, LF = laminectomy and fusion, M–H = Mantel–Haenszel.

Twenty-three studies^[20–22,26,30,34,44,46,48,49,56,60,61,66,73,81,83,84,88,89,92,118,119]^ containing 372 patients with axial pain of 2650 patients after cervical surgery were included for meta-analysis. Figure 25 shows that the incidence was 15.6% (95% CI 11.7%–19.5%), without substantial heterogeneity of incidence observed. The incidence varied between 1.7% and 53.2%. Incidence of axial pain for those following LP and LF was 22.2% (95% CI 14.1%–29.3%) and 23.2% (95% CI 15.8%–31.3%) (Figs. 26, 27).

### 3.9 Hoarseness

**Figure 28:**
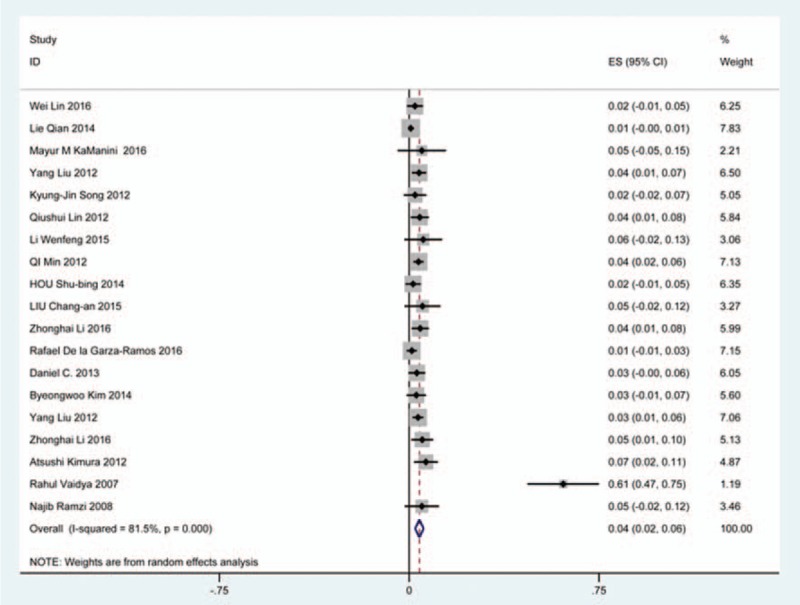
Forest plot showing incidence of hoarseness. CI = confidence interval, df = degrees of freedom, M–H = Mantel–Haenszel.

**Figure 29:**
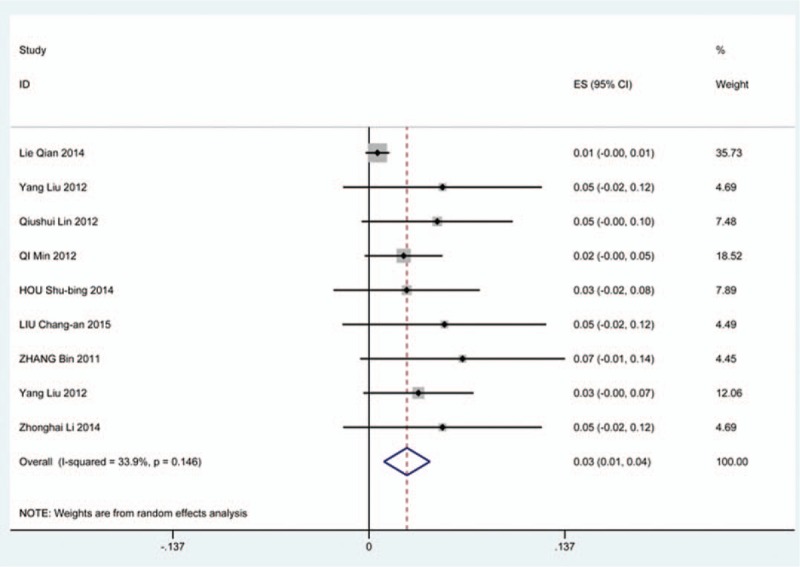
Forest plot showing incidence of hoarseness after ACCF. ACCF = anterior cervical corpectomy and fusion, CI = confidence interval, df = degrees of freedom, M–H = Mantel–Haenszel.

**Figure 30:**
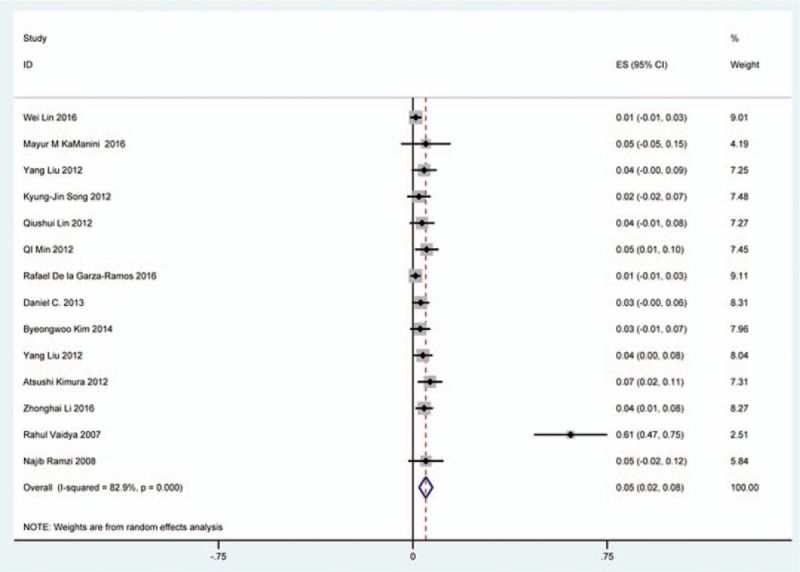
Forest plot showing incidence of hoarseness after ACDF. ACDF = anterior cervical discectomy and fusion, CI = confidence interval, df = degrees of freedom, M–H = Mantel–Haenszel.

Nineteen studies^[25,26,31,35,36,38,40,45,56,62,70,79,81,84–86,90,100,116]^ containing 99 patients with hoarseness of 2234 patients after cervical surgery were included. Figure 28 shows that the incidence of hoarseness was 4.0% (95% CI 2.3%–5.7%), with substantial heterogeneity of incidence observed. The incidence varied between 0.6% and 60.9%. Patients after ACDF (4.8%, 95% CI 1.9%–7.8%) had a slight higher prevalence of hoarseness than those with ACCF (3.0%, 95% CI 0.9%–4.2%) (Figs. 29, 30).

### 3.10 Epidural hematoma

**Figure 31:**
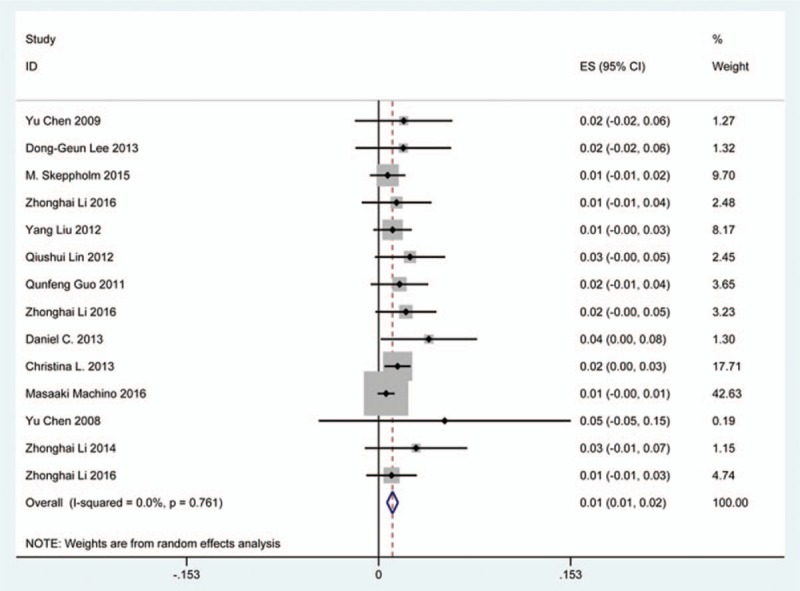
Forest plot showing incidence of epidural hematoma. CI = confidence interval, df = degrees of freedom, M–H = Mantel–Haenszel.

**Figure 32:**
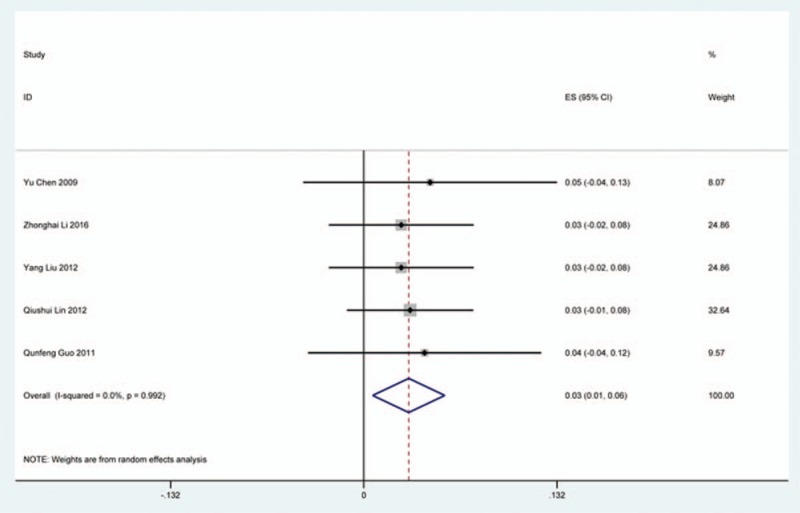
Forest plot showing incidence of epidural hematoma after ACCF. ACCF = anterior cervical corpectomy and fusion, CI = confidence interval, df = degrees of freedom, M–H = Mantel–Haenszel.

**Figure 33:**
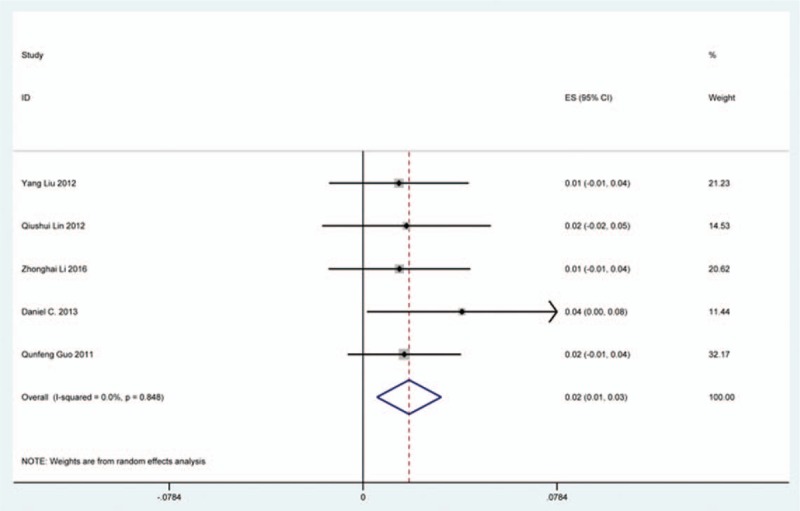
Forest plot showing incidence of epidural hematoma after ACDF. ACDF = anterior cervical discectomy and fusion, CI = confidence interval, df = degrees of freedom, M–H = Mantel–Haenszel.

Fourteen studies^[22,23,33–35,38,39,56,61,65,74,79,81,120]^ containing 33 patients with epidural hematoma of 2185 patients after cervical surgery were included. Figure 31 shows that the incidence was 1.1% (95% CI 0.7%–1.5%), without substantial heterogeneity of incidence observed. The incidence varied between 0.5% and 5.3%. Incidence of axial pain for those following ACCF and ACDF was 3.1% (95% CI 1.0%–6.2%) and 2.0% (95% CI 0.9%–3.2%) (Figs. 32, 33).

### 3.11 Graft dislodgment

**Figure 34:**
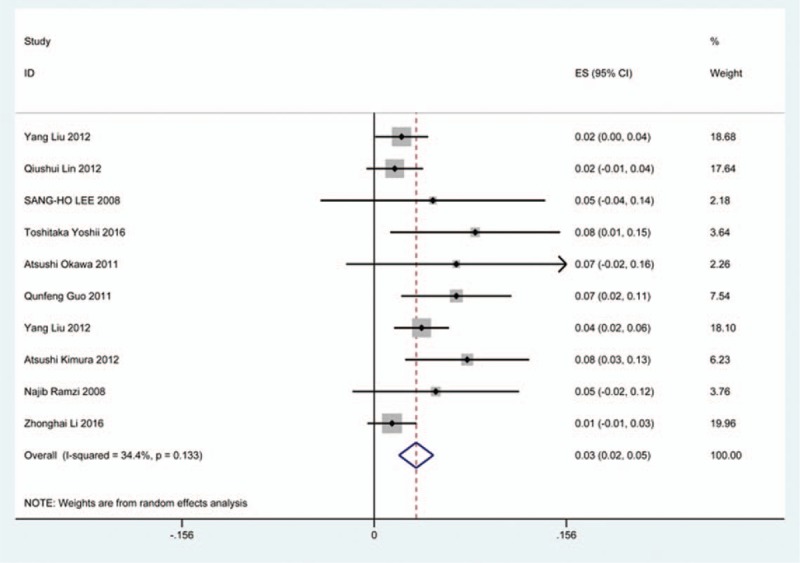
Forest plot showing incidence of graft dislodgment. CI = confidence interval, df = degrees of freedom, M–H = Mantel–Haenszel.

Ten studies^[35,38,40,45,62,64,69,81,82,121]^ containing 45 patients with graft dislodgment of 1102 patients after cervical surgery were included. Figure 34 shows that the incidence was 3.4% (95% CI 2.0%–4.8%), without substantial heterogeneity of incidence observed. The incidence among the studies varied between 1.4% and 8.2%.

### 3.12 Graft subsidence

**Figure 35:**
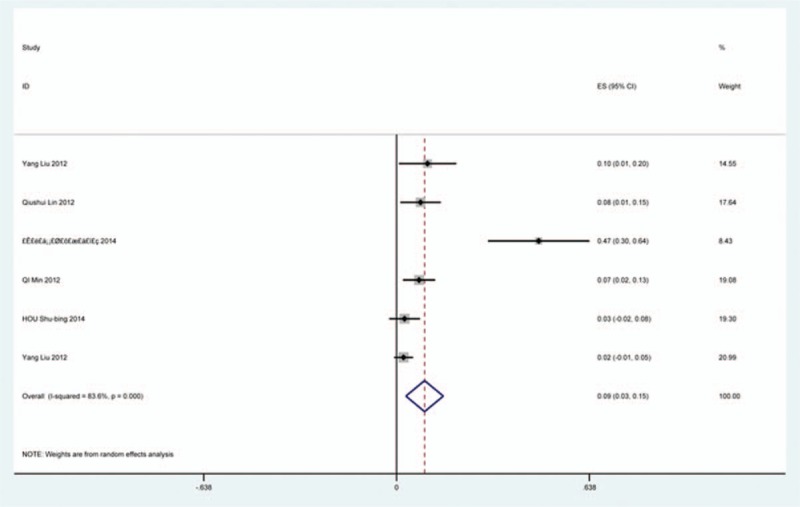
Forest plot showing incidence of graft subsidence. CI = confidence interval, df = degrees of freedom, M–H = Mantel–Haenszel.

Six studies^[34,35,38,39,84,115]^ containing 26 patients with graft subsidence of 591 patients after cervical surgery were included. Figure 35 shows that the incidence of graft subsidence was 3.7% (95% CI 2.0%–5.5%), with substantial heterogeneity of incidence observed. The incidence varied between 2.2% and 11.1%.

### 3.13 Fusion failure

**Figure 36:**
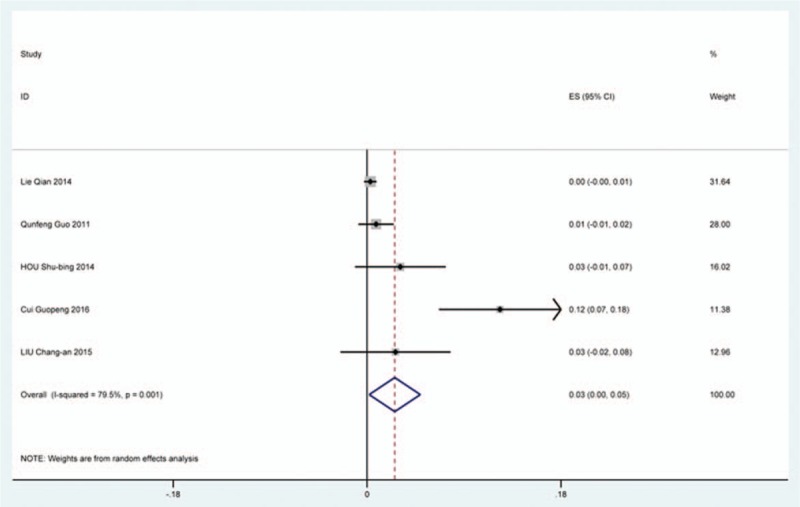
Forest plot showing incidence of fusion failure. CI = confidence interval, df = degrees of freedom, M–H = Mantel–Haenszel.

Five studies^[26,39,86,87,90]^ containing 21 patients with fusion failure of 689 patients after cervical surgery was included. Figure 36 shows that the incidence was 2.6% (95% CI 0.2%–4.9%), with substantial heterogeneity of incidence observed. The incidence varied between 0.2% and 12%.

### 3.14 Publication bias

**Figure 37:**
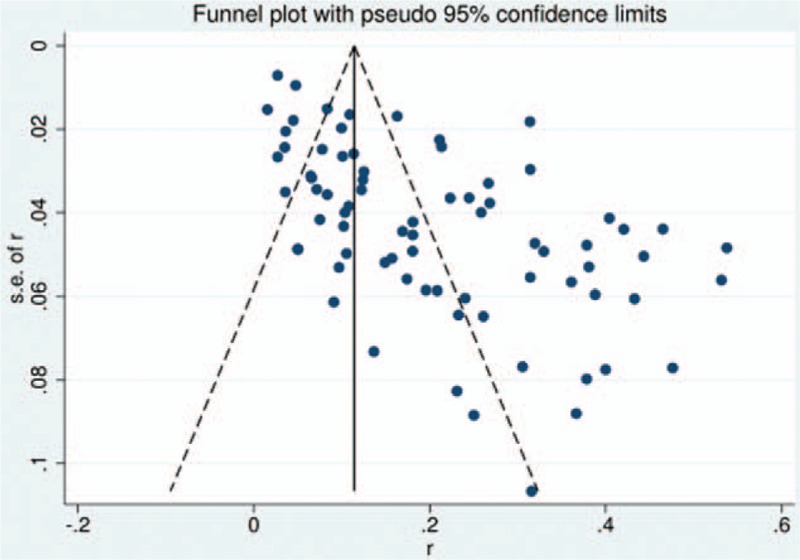
Funnel plot showing incidence of all complications after cervical surgery.

We performed funnel plot for publication bias, as shown in Fig. 37, after a detection of publication bias by Egger and Begg tests using STATA 12.0, there was no publication bias found for all included studies (all *P* > 0.05).

## 4 Discussion

Increasing studies focused on surgical selection for cervical compressive myelopathy (CCM), which usually caused by CSM or OPLL.^[3–5,33]^ Nearly half a century, surgical procedures were widely applied from posterior approaches including LF and LP to anterior approaches containing ACDF, ACCF, and ACCDF.^[47,78]^ Nevertheless, the option of surgical approach remains debated. Especially, the inevitable complications of anterior and posterior approaches cause our attention.^[22,36,48]^ Anterior approaches had a higher rate of postoperative hoarseness, dysphagia.^[55,81]^ Similarly, C5 palsy and cervical kyphosis may limit the use of posterior surgery.^[26]^ The complications in our study included overall complications, C5 plasy, cerebrospinal fluid (CSF), infection, axial pain, dysphagia, hoarseness, fusion failure, graft subsidence, graft dislodgment, and epidural hematoma. As we know, this is the first meta-analysis on prevalence of various complications after cervical surgery. The aim of the study is to compute prevalence of each complication according to previous studies. We hope that our work can give some suggestions to assess incidence of complications before surgery.

Our results showed that the rates for total complications, C5 plasy, CSF, infection, axial pain, dysphagia, hoarseness, fusion failure, graft subsidence, graft dislodgment and epidural hematoma were 20.1%, 5.3%, 1.9%, 2.8%, 15.6%, 16.8%, 4.0%, 2.6%, 3.7%, 3.4%, 1.1%, respectively. Compared with patients with CSM, patients with OPLL have a higher incidence of C5 plasy (4.1% vs 6.3%) and CSF (0.9% vs 12.2%). In terms of C5 plasy, patients after LP had the highest rate (15.2%), while those after ACDF had the lowest rate (2.0%). As for dysphagia, patients who underwent ACCDF and ACDF were 16.8% and 16.2%, which are higher than those who received ACCF (9.9%). For CSF, patients who underwent ACCF had the highest rate (4.2%), while those who received ACDF had the lowest rate (1.9%), and the same trend for infection between ACCF group (14.2%) and ACDF group (0.9%). While it was opposite for hoarseness between ACDF (4.8%) and ACCF (3.0%).

A number of studies focused on the occurrence of C5 palsy after cervical surgery. Even though some mechanisms trying to explain this common complication have been proposed, it remained a controversial issue. C5 palsy after cervical surgery is considered to be a result of nerve root injury or segmental spinal cord disorder.^[36–41,51–55]^ We reviewed 57 studies and the rate of C5 plasy was 5.3%. We also found that patients with CSM (4.1%) have a lower incidence of C5 palsy than patients with OPLL (6.3%). The reason is still unclear. In all surgical options, LF had the highest rate, ACDF had the lowest incidence. Nakashima^[72]^ reported that C5 palsy was caused by posterior shift of the spinal cord, and additional iatrogenic foraminal stenosis due to cervical alignment correction after posterior instrumentation with fusion. It was obvious that posterior shift of the spinal cord in LF was largest, which was similar to our results.

Dysphagia is a relatively common complication after cervical surgery. Smith-Hammond et al^[121]^ found that the prevalence of dysphagia on the first postoperative day was approximately 50% in the anterior cervical group. As Fig. 11 has shown, the rate was 16.8% (95% CI 13.6%–19.9%). According to included articles in our series, the rate for this complication ranged from 1.4% to 58.1%. Patients after ACCDF (16.8%, 95% CI 6.9%–27.2%) and ACDF (16.2%, 95% CI 11.7%–19.8%) had higher incidence than those who received ACCF (9.9%, 95% CI 4.8%–15.9%). Multifactors as reported by recent studies,^[63,68–71,75–80]^ hematoma, pharyngeal plexus denervation, vocal cord paralysis, adhesion formation, plate profile, and swelling due to biologic agents, may be related to dysphagia. Brad^[99]^ indicated that a no-profile cervical disc arthroplasty had a significantly lower rate of dysphagia compared with ACDF. Due to few included articles on disc arthroplasty, we did not assess rate of dysphagia in arthroplasty group.

CSF is a serious complication of cervical surgery,^[87,88]^ which may cause wound infection, purulent meningitis, or even high risk of death. Rate of CSF after cervical operation ranged from 0.4% and 21.1%^[122]^. As the same with previous reports, our results implied that patients with OPLL had a higher rate compared with those with CSM. We surprisingly found that patients after ACCF (4.2%) had a higher rate than those who received ACDF (1.9%), which was possibly different with our thinking. Compared with ACCF, operative field of ACDF was smaller, which was more likely to cause CSF. Large sample studies are needed to further investigate this issue. Figure 21 shows that the rate of infection was 2.8%. The same trend as CSF, individual who underwent ACCF (14.2%) had higher than those who received ACDF (0.9%) and LP (2.1%).

As for axial pain, which is terrible complication after posterior approaches, the results show that LP (22.2%) and LF (23.2%) are similar. Muscles were widely dissected and ligamentous structures transected in both techniques resulting in axial pain to some extent. Hoarseness and epidural hematoma had relatively low rate. Overstretch or improper handing may lead to these complications. We also estimated implant-related complications, but the rate on these complications in various surgical options were not assessed due to lack relevant data. The rates of graft dislodgment, graft subsidence, and fusion failure were 3.7%, 3.4%, and 1.1%.

There are several limitations of this study. First, there was no RCT on all complications, we need RCT to further study; second, the statistical power could be improved in the future by including more studies. Some parameters, like one-level, two-level, or multilevel CSM for C5 palsy, due to lack of data could not be analyzed by subgroups to avoid a high heterogeneity which may exert instability on the consistency of the outcomes; third, the searching strategy was restricted to articles published in the English and Chinese languages. Articles with potentially high-quality data published in other languages were not included because of anticipated difficulties in obtaining accurate medical translations. Fourth, it is difficult to avoid that many figures presented high heterogeneity due to relative large sample.

In summary, the rate of overall complications was 21%, patients with OPLL have a higher incidence of C5 palsy and CSF. Patients after LF have a higher incidence of C5 palsy, ACCDF have a higher incidence of dysphagia, ACCF have a higher incidence of CSF and infection, and ACDF have a higher incidence of hoarseness. Considering the limitations noted above, a further well-designed, large population-based study on the topic of the prevalence of complications after cervical surgery should be conducted.
